# Factors that influence early breastfeeding of singletons and twins in Korea: a retrospective study

**DOI:** 10.1186/s13006-016-0094-5

**Published:** 2017-01-07

**Authors:** Bo-Yeoul Kim

**Affiliations:** College of Nursing, Eulji University, 77 Gyeryong-ro 771 beon-gil, Jung-gu, Daejeon, 301-746 South Korea

**Keywords:** Breastfeeding, Twins, Newborn, Feeding methods, Lactation, Seoul, Korea

## Abstract

**Background:**

This study aimed to evaluate the factors that influence breastfeeding throughout the hospital stay, including prenatal preparation, early skin-to-skin contact directly after delivery, rooming-in, feeding before the first breastfeed, time to first breastfeed, and postpartum support. This study also aimed to verify whether these factors were significant after adjusting for the mother’s characteristics, the newborn’s characteristics, and the delivery characteristics.

**Methods:**

A retrospective survey was used to collect the data. Factors that influenced breastfeeding throughout the hospital stay, and differences between the types of newborns were analyzed.

**Results:**

Among twins, a higher likelihood of breastfeeding throughout the hospital stay was associated with not feeding before the first breastfeeding and an earlier start time for the first breastfeeding. Among singletons, a higher likelihood of breastfeeding throughout the hospital stay was associated with early skin-to-skin contact, no other feeding before the first breastfeed, and an earlier commencement of the first breastfeed.

**Conclusions:**

Effort should encourage early breastfeeding, without restriction, to improve the breastfeeding rate among mothers of twins. Moreover, an individualized approach that addresses the factors that influence breastfeeding for each type of newborn may help improve the corresponding rates of breastfeeding throughout the hospital stay.

## Background

The frequency of multiple births is gradually increasing worldwide [1, 2], and this increase may be related to the number of mothers who conceive after the age of 30 years, as well as the spread of various fertility treatments [3]. For example, the proportion of twins in the US has increased every year since 1990. In Korea, the proportion of new mothers who are > 35 years old has increased from 2.1% in 1985 to 8.5% in 2003 [4] and to 21.6% in 2012 [5]. Similarly, the proportion of multiple births among Korean mothers of childbearing age has increased from 1.5% in 1998 to 2.7% in 2010 [6].

Although the World Health Organization and United Nations International Children’s Fund suggest that a mother’s milk is sufficient for feeding multiple babies [7], many mothers experience difficulties in the clinical setting and with early breastfeeding [8, 9]. Furthermore, the breastfeeding rate for twins is lower than that for singleton babies [8, 10]. The Japanese rates of exclusive breastfeeding were 42.2% at one month and 38% at three months after delivery in 2005 [11, 12]. However, the Korean rates of exclusive breastfeeding were 65.6% at one month and 57% at three months after delivery in 2009 [13]. Among mothers who wanted to breastfeed but did not practice breastfeeding, 60.1% reported the lack of opportunity to breastfeed during their post birth hospitalization [14].

It is recommended that breastfeeding be initiated within 30–60 minutes after birth to promote successful breastfeeding [7]. Thus, the initial feeding should occur immediately after birth while the infant is awake, alert, and ready to suckle. Catecholamines are rapidly secreted by the newborns during the first two hours after birth [15], and the early initiation of breastfeeding after birth will help the baby imprint on the mother’s milk. Newborns exhibit an imprint phenomenon whereby they remember the taste, smell, and feel associated with breastfeeding, and it is recommended that babies should only be fed mother’s milk directly from the breast [7]. However, the in-hospital use of supplementary bottles and teats may increase the infant’s preexisting breastfeeding difficulty and magnify the mother’s perception of that difficulty [16]. Bottle feeding can also create a serious obstacle for breastfeeding, as it can reduce the mother’s milk supply. Some researchers believe it may also cause nipple confusion in the newborn, as they may become used to the bottle teat and may exhibit confusion when attempting to feed at the breast [17]. Thus, keeping the mother and infant(s) together (“rooming-in”) at all times help improve breastfeeding success [18]. An abundance of readily available premixed formula bottles may encourage healthcare providers to recommend their use, although this approach is not in the best interest of breastfeeding women and their babies.

A mother’s decision to breastfeed her baby is usually made before or during the pregnancy, and mothers who make the decision before pregnancy are more likely to sustain breastfeeding. Therefore, an effort made during the early antenatal period to include fathers in supporting the breastfeeding process [19]. Breastfeeding mothers require guidance, education, and support during the immediate postpartum period and during the first few weeks while they establish breastfeeding. Unfortunately, the efforts of healthcare providers to support breastfeeding can be undermined by a variety of factors, including hospital practice, the maternity setting, and social barriers [20]. Mothers who deliver multiple babies often experience difficulty with the initiation of breastfeeding during their hospitalization, and appropriate interventions and support are needed [21]. Furthermore, the birth of twin babies is physically and mentally demanding compared to a singleton birth, and is associated with numerous obstacles to breastfeeding [22]. Thus, systematic support and education by healthcare professionals will ensure that the mother has initiated breastfeeding and an adequate milk production [23], and it would be useful to identify factors that influence early breastfeeding of twins throughout the mother’s hospitalization.

Nipple confusion describes a neonate’s difficulty, after exposure to a bottle teat, in achieving the correct oral configuration, latching technique, and suckling pattern that are necessary to extract milk from the breast. This phenomenon typically occurs when an artificial teat is introduced before the successful establishment of breastfeeding. It is especially likely to occur when sucking the bottle teat results in a more rapid flow of fluid, compared to the flow of breast milk from the mother’s breast [24]. In addition, sore nipples can limit the time breastfeeding, although this complication is most frequently caused by incorrect latching or positioning, rather than the act of breastfeeding multiples [25]. Avoiding the use of artificial teats for breastfed newborns is an integral component of the World Health Organization/UNICEF Baby Friendly Hospital Initiative, which is a global campaign that aims to remove obstacles to breastfeeding that a woman may encounter in a hospital [8]. Early breastfeeding promotes lactogenesis II a few days after birth, which is when the mother’s milk supply changes from a small volume of colostrum to larger volumes of mature milk [26].

Although previous studies have examined breastfeeding throughout the hospital stay for singletons, only a small number of studies have examined breastfeeding throughout the hospital stay for twins [8, 10]. Moreover, the frequency of multiple births is increasing in Korea. Therefore, the present study examined cases from a Korean hospital and evaluated various factors that influenced breastfeeding throughout the hospital stay among mothers of singletons and twins.

### Hypothesis

Breastfeeding among mothers of singletons and twins throughout the postpartum hospital stay is influenced by prenatal preparation, early skin-to-skin contact after delivery, first feeding factors (feeding before the first breastfeeding and time to first breastfeeding), rooming-in, and breastfeeding support.

## Methods

### Study Design

This retrospective comparative study carried out at the Seoul National University Hospital, included mothers who gave birth and breastfed their babies (singletons or twins) for approximately three days after a vaginal birth and approximately one week after a Cesarean section. Mothers who could not continue to breastfeed for medical reasons were excluded. In addition, mothers with three or more surviving babies and babies with a low birth weight (<1.5 kg) were excluded. The comparative component of the study compared the factors that influenced early breastfeeding of singletons and twins.

A survey of mothers who gave birth at the Seoul National University Hospital in 2011 was used to check the breastfeeding rate among women who breastfed throughout the hospital stay. Using that rate, with a power of 80% and a significance level of 5%, the required sample size for hypothesis testing was 208 women and their babies (104 cases for each group).

### Data collection

#### Survey regarding postpartum support

Phone surveys of mothers who had breastfed their babies during the first post-birth week revealed a response rate of approximately 20%. Independent statistical experts used the PROC SURVEYSELECT feature of SAS software to create a random sample of 1,873 mothers (382 mothers of twins and 1,491 mothers of singletons) who had given birth in hospital between April 9, 2009 and October 1, 2013. The response rate for the phone survey (January 3, 2014 to February 28, 2014) was 20.1% (376 mothers, including 130 mothers of twins and 246 mothers of singletons). After performing the PROC SURVEYSELECT process, I tried to make the sample of mothers of singletons proportional to the sample of mothers of twins (382 mothers of twins vs 1,491 mothers of singletons). However, I included all mothers who responded for this study, and I assumed that mothers with twins would participate in the survey, because they were likely to receive antepartum/intrapartum/postpartum therapy and breastfeeding instruction during their hospitalization and they likely intended to visit outpatient clinics after their hospitalization. Thus, selection bias may be present, although I do not believe that it significantly influenced the findings.

The study sample was deemed adequate for the analyses, and each mother was contacted by phone at least three times in an attempt to achieve a high response rate. However, the relatively low response rate was primarily related to personal circumstances, such as baby care, work, and concerns regarding privacy issues. Data from the non-responders were not available, and it was not possible to compare the mothers who did and did not respond to the survey.

#### Medical record audit

Using the mothers’ serial numbers, data were retrieved from their electronic medical records, and data regarding breastfeeding and supplementary feeding during the hospitalization were obtained using a data collection form. In cases with twins, the first-born newborn was selected for analysis. Furthermore, 10 mothers were excluded because they could not continue to breastfeed for medical reasons, or because the hospital records did not provide complete data regarding breastfeeding. Therefore, 366 mothers were included in the final analyses (241 mothers of singletons and 125 mothers of twins).

### Data measures

The present study evaluated various factors that influenced breastfeeding throughout the hospital stay of singletons and twins, such as prenatal preparation, early skin-to-skin contact directly after delivery, rooming-in, feeding before the first breastfeeding, time to first breastfeeding, and postpartum support [15].

‘Breastfeeding throughout the hospital stay’ was defined as Stages I–II from the World Health Organization’s five stages of infant nutritional classification [27], which address breastfeeding frequency, daily intake, and supplemental feeding (sugar water or infant formula) throughout the hospital stay. ‘Early skin-to-skin contact’ was defined as the placing of the naked baby prone on the mother’s bare chest within 30 minutes after delivery, and ‘the first breastfeeding’ was defined as the baby suckling directly at the breast for the first time after birth [15]. ‘Feeding before the first breastfeeding’ was defined as supplemental feeding (sugar water or infant formula) that was provided to the infant before any breastfeeding [7].

### Statistical analysis

The normality of continuous variables were assessed using a Q-Q plot and the Kolmogorov-Smirnov test. Normally distributed continuous data were reported as mean ± standard deviation, non-normally distributed data were reported as median [minimum, maximum], and categorical data were reported as number and frequency. The comparisons between the groups that did and did not practice breastfeeding was performed using the independent t-test or Mann–Whitney U test for continuous data, and using the χ^2^ -test or Fisher’s exact test for non-continuous data. A multivariable logistic regression model was used to evaluate the independent relationships between the factors and breastfeeding throughout the hospital stay. Factors with a *p*-value of ≤ 0.2 from Tables 1, 2 and 3 were selected as the control variables for the multivariable model. The non-modifiable factors that could influence breastfeeding practice [28] were defined as the mother's age, residential area, occupation, education, gestational period, parity, delivery mode, type of anesthesia, newborn’s sex, weight at birth, Apgar score (after 5 minutes), and health status. The modifiable factors were early skin-to-skin contact, rooming-in, feeding before the first breastfeeding, time to first breast milk feeding after birth, prenatal preparation, husband’s support, and other family members’ support [15, 29]. Although rooting reflex and sucking reflex were significant factors in Table 2, they were not included as control variables in the multivariable model because the sample size was too small and because they exhibited collinearity with Apgar score and health status. Thus, including these factors as control variables would not change the adjusted odds ratios (ORs).

**Table 1:** Mothers’ characteristics (*n* = 366)

Characteristic	*n*	Categories	Total	Singletons (*n* = 241)			Twins (*n* = 125)		
				No practice group	Practice group	*p-*value	No practice group	Practice group	*p*-value
			Median [Min, Max] or *n* (%)	Median [Min, Max] or *n* (%)	Median [Min, Max] or *n* (%)		Median [Min, Max] or *n* (%)	Median [Min, Max] or *n* (%)	
Age (years)			31 [20, 42]	32 [22, 42]	30 [20, 41]	0.148^†^	30 [20, 41]	29 [20, 41]	0.455^†^
Residential area	366	Seoul capital area	327 (89.3)	110 (85.3)	103 (92.0)	0.282^§^	73 (94.8)	41 (85.4)	0.052^§^
		Other	39 (10.7)	19 (14.7)	9 (8.0)		4 (5.2)	7 (14.6)	
Occupation status	363	Housewife	209 (57.6)	78 (61.4)	59 (52.7)	0.173^‡^	46 (60.5)	26 (54.2)	0.485^‡^
		Employed	154 (42.4)	49 (38.6)	53 (43.7)		30 (39.5)	22 (45.8)	
Education status	361	≤ High school	50 (13.9)	25 (19,7)	14 (12.6)	0.189^‡^	6 (8.0)	5 (10.4)	0.676^‡^
		College	261 (72.2)	85 (66.9)	86 (77.5)		57 (76.0)	33 (68.8)	
		≥ Graduate school	50 (13.9)	17 (13.4)	11 (9.9)		12 (16.0)	10 (20.8)	
Iron during pregnancy	333	Yes	319 (95.8)	106 (94.6)	98 (96.1)	0.751^§^	70 (95.9)	45 (97.8)	0.999^§^
		No	14 (4.2)	6 (5.4)	4 (3.9)		3 (4.1)	1 (2.2)	
Folate during pregnancy	333	Yes	57 (17.1)	18 (16.1)	19 (18.6)	0.621^‡^	13 (17.8)	7 (15.2)	0.713^‡^
		No	276 (82.9)	94 (83.9)	83 (81.4)		60 (82.2)	39 (84.8)	

^†^Mann–Whitney U test, ^‡^chi-square test, ^§^Fisher’s exact test, Practice group: Breastfed throughout the hospital stay, No practice group: Not breastfed throughout the hospital stay

**Table 2:** Newborns’ characteristics (*n* = 366)

Characteristics	*n*	Categories	Total	Singletons (*n* = 241)			Twins (*n* = 125)		
				No practice group	Practice group	*p*-value	No practice group	Practice group	*p*-value
			Mean ± SD Median [Min, Max] or *n* (%)	Mean ± SD Median [Min, Max] or *n* (%)	Mean ± SD Median [Min, Max] or *n* (%)		Mean ± SD Median [Min, Max] or *n* (%)	Mean ± SD Median [Min, Max] or *n* (%)	
Birth order	362	1^st^	239 (66.0)	76 (58.9)	68 (60.7)	0.99^§^	55 (73.3)	40 (86.9)	0.031^§^
		2^nd^	92 (25.4)	38 (29.5)	31 (27.7)		19 (25.3)	4 (8.7)	
		≥3^rd^	31 (8.6)	15 (11.6)	13 (11.6)		1 (1.4)	2 (4.4)	
Sex	366	Male	199 (54.4)	77 (59.7)	55 (49.1)	0.01^‡^	41 (53.3)	26 (54.2)	0.92^‡^
		Female	167 (45.6)	52 (40.3)	57 (50.9)		36 (46.7)	22 (45.8)	
Weight (kg)	366		2.98 [1.63, 4.72]	3.17 [1.93, 4.72]	3.31 [2.05, 4.14]	0.018^†^	2.57 [1.63, 3.48]	2.56 [2.03, 3.18]	0.198^†^
Age at discharge (hours)	366		271.9 [232.5, 325]	276 [232.5, 315]	277.95 [248, 325]	0.675^†^	263.9 [244, 274.9]	264.85 [247.7, 282]	0.128^†^
Gestational period (weeks)	365	<36	36 (9.8)	9 (7.0)	1 (0.9)	0.059^‡^	22 (28.6)	4 (8.3)	0.018^‡^
		36–37	98 (26.7)	22 (17.0)	13 (11.7)		33 (42.8)	30 (62.5)	
		38–39	166 (45.8)	66 (51.2)	64 (57.6)		22 (28.6)	14 (29.2)	
		>40	65 (17.7)	32 (24.8)	33 (29.7)		0 (0.00)	0 (0.00)	
Apgar score	365	<6	8 (2.2)	5 (3.9)	2 (1.8)	<0.001^§^	1 (1.3)	0 (0.00)	0.789^§^
(after 5 min)		6–8	80 (21.9)	48 (37.2)	5 (4.5)		18 (23.7)	9 (18.7)	
		>9	277 (75.9)	76 (58.9)	105 (93.7)		57 (75.0)	39 (81.3)	
Health status	350	Abnormal	29 (8.3)	17 (15.0)	3 (2.7)	0.001^‡^	6 (7.8)	3 (6.2)	0.999^§^
		Normal	321 (91.7)	96 (85.0)	109 (97.3)		71 (92.2)	45 (93.8)	
Rooting reflex	344	Good	329 (95.6)	105 (87.5)	112 (48.27)		65 (100)	47 (100)	
		Moderate	15 (4.4)	14 (11.7)	0 (0.00)	<0.001^§^	0 (0.00)	0 (0.00)	-
		Bad	1 (0.3)	1 (0.8)	0 (0.00)		0 (0.00)	0 (0.00)	
Sucking reflex	345	Good	330 (95.7)	105 (87.5)	112 (100)		65 (100)	48 (100)	
		Moderate	14 (4.0)	14 (11.7)	0 (0.00)	<0.001^§^	0 (0.00)	0 (0.00)	-
		Bad	1 (0.3)	1 (0.8)	0 (0.00)		0 (0.00)	0 (0.00)	

Independent t-test, ^†^Mann–Whitney U test, ^‡^chi-square test, ^§^Fisher’s exact test. Practice group: Breastfed throughout the hospital stay, No practice group: Not breastfed throughout the hospital stay

**Table 3:** Delivery characteristics (*n* = 366)

Characteristics	*n*	Categories	Total	Singletons (*n* = 241)			Twins (*n* = 125)		
				No practice group	Practice group	*p*-value	No practice group	Practice group	*p*-value
			*n* (%)	*n* (%)	*n* (%)		*n* (%)	*n* (%)	
Delivery mode	366	Vaginal delivery	251 (68.6)	90 (69.8)	81 (72.3)	0.663^‡^	48 (62.3)	32 (66.7)	0.624^‡^
		Cesarean section	115 (31.4)	39 (30.2)	31 (27.7)		29 (37.7)	16 (33.3)	
Type of anesthesia	366	Local	251 (68.6)	90 (69.8)	81 (72.3)	0.856^§^	48 (62.3)	32 (66.7)	0.96^§^
		Epidural	23 (6.3)	5 (3.9)	6 (5.4)		8 (10.4)	4 (8.3)	
		General	13 (3.5)	4 (3.1)	3 (2.7)		4 (5.2)	2 (4.2)	
		Spinal	79 (21.6)	30 (23.2)	22 (19.6)		17 (22.1)	10 (20.8)	

Mann–Whitney U test, ^‡^chi-square test, ^§^Fisher's exact test, Practice group: Breastfed throughout the hospital stay, No practice group: Not breastfed throughout the hospital stay

The 0.2 level of significance to select control variables was based on the statistical perspective, as univariate analyses for selecting control variables might fail to identify important variables at the conventional level (0.05). Thus, it is advisable to set the significance level much higher than the conventional levels (i.e. ≥ 0.2) [30]. All statistical analyses performed used SAS software (version 9.2).

### Ethical considerations

This study was approved by the institutional review board of the Seoul National University College of Medicine and Seoul National University Hospital (H-1306-095-499). All participants provided their informed consent to participate in the phone survey portion of the study and for their responses to be recorded.

## Results

The mothers’ , newborns’ , and delivery characteristics, as well as the factors influencing breastfeeding throughout the hospital stay, are summarized in Tables 1, 2, Table 3 and 4. The overall rate of breastfeeding throughout the hospital stay was 43.7% (38.4% for twins and 46.5% for singletons), which was not significantly different from the rate of 47.1% for all newborns who were born during 2008 at the Seoul National University Hospital. The average age of the mothers was 31 years (range: 20–42 years). Most participants lived in Seoul (89.3%), were housewives (57.6%), and had graduated college (72.2%). Smaller proportions of the mothers had completed graduate school (13.9%) or high school (13.9%) (Table 1). The proportions of the newborns’ birth order were 66% for the first delivery, 25.4% for the second delivery, and 8.6% for the third or greater delivery. Twins had an average weight of 2.58 ± 0.37 kg, and singletons had a larger average weight of 3.19 ± 0.49 kg.

**Table 4:** Factors that influenced breastfeeding (*n* = 366)

Characteristics	*n*	Categories	Total	Singletons (*n* = 241)				Twins (*n* = 125)	
				No practice group	Practice group	*p*-value	No practice group	Practice group	*p*-value
			Mean ± SD Median [Min, Max] or *n* (%)	Mean ± SD Median [Min, Max] or *n* (%)	Mean ± SD Median [Min, Max] or *n* (%)		Mean ± SD Median [Min, Max] or *n* (%)	Mean ± SD Median [Min, Max] or *n* (%)	
Type of newborn	366	Singleton	241 (65.9)	-	-	-	-	-	-
		Twin	125 (34.1)						
Early skin-to-skin contact	359	Yes	284 (79.1)	79 (62.2)	105 (96.3)	<0.001^¶^	56 (74.7)	44 (91.7)	0.018^¶^
		No	75 (20.9)	48 (37.8)	4 (3.7)		19 (25.3)	4 (8.3)	
Time to early skin-to-skin contact (min)	286		9 [1, 29]	10 [1, 29]	9 [3, 13]	0.831^∥^	9 [5, 22]	8 [4, 25]	0.338^∥^
Rooming in	328	Yes	273 (83.2)	89 (73.5)	93 (90.3)	0.001^¶^	55 (84.6)	36 (92.3)	0.251^¶^
		No	55 (16.8)	32 (26.5)	10 (9.7)		10 (15.4)	3 (7.7)	
Feeding before the first breastfeed	366	Yes	132 (36.1)	75 (58.1)	12 (10.7)	<0.001^#^	40 (52.0)	5 (10.4)	<0.001^¶^
		No	234 (63.9)	54 (41.9)	100 (89.3)		37 (48.0)	43 (89.6)	
Time to first BMF (hours)	309		6.62 [0.7, 111.1]	22.95 [4.1, 111.1]	6.23 [2.67, 41.33]	<0.001^∥^	6.67 [0.7, 104.18]	6.17 [3.35, 24.77]	0.117^∥^
Prenatal preparation	366		1 [0, 3]	1 [0, 3]	1 [1, 3]	0.283^∥^	1 [1, 3]	1 [0, 3]	0.522^∥^
Satisfaction with husband’s support	357		2 [1, 4]	2 [1, 4]	2 [1, 4]	0.716^∥^	2 [1, 4]	2 [1, 4]	0.809^∥^
Satisfaction with other family members’ support	354		3 [1, 4]	3 [1, 4]	3 [1, 4]	0.907^∥^	3 [1, 4]	3 [1, 4]	0.385^∥^

Independent t-test, ^∥^Mann–Whitney U test, ^¶^chi-square test, ^#^Fisher’s exact test

BMF: breast milk feeding, Practice group: Breastfed throughout the hospital stay, No practice group: Not breastfed throughout the hospital stay, Early: within 30 minutes after the delivery

Prenatal preparation was scored using the following scale: 0 = not prepared, 1 = less prepared, 2 = prepared, and 3 = well prepared

Satisfaction with husband’s support and with other family members’ support was scored using the following scale: 1 = very dissatisfied, 2 = dissatisfied, 3 = satisfied, 4 = very satisfied

The gestational period was 38–39 weeks for 45.8% of the newborns and 36–37 weeks for 26.7% of the newborns. Approximately 71.2% of the twins had a gestational period of ≤37 weeks, compared to approximately 18.7% of the singletons (Table 2).

The twins exhibited frequencies of 64% for vaginal delivery and 36% for Cesarean section, compared to 71% and 29%, respectively, among singleton births. Among singletons, the rate of breastfeeding throughout the hospital stay rate was 47% for vaginal delivery and 44% for Cesarean section, while the rates among twins were 40% and 36%, respectively. The delivery mode was not significantly associated with breastfeeding throughout the hospital stay among singletons or twins (both, *p* > 0.5). However, a vaginal delivery was associated with a 3–4% higher breastfeeding rate (vs. Cesarean section) among both singletons and twins, although the differences were not statistically significant (both, *p* > 0.5) (Table 3).

The rate of rooming-in was 83.2%. Early skin-to-skin contact was documented in 79.1% of the cases, and began at an average of nine minutes after birth (range: 1 to 29 minutes). The incidence of feeding before the first breastfeeding was 36.1%, (Table 4).

After adjusting for the variables that were expected to influence breastfeeding, twins who were not fed before the first breastfeeding exhibited an increased likelihood of breastfeeding throughout the hospital stay (OR 7.684, 95% confidence interval [CI] 2.587, 22.817), compared to twins who received supplemental food (*p* < 0.001). Each hour delay in the first breastfeeding session for twins was associated with a reduced likelihood of breastfeeding throughout the hospital stay (OR 0.916, 95% CI 0.856, 0.98, *p* = 0.011), and this relationship was observed for singletons (OR 0.886, 95% CI 0.833, 0.942, *p* < 0.001). Rooming-in was associated with an increased likelihood of breastfeeding throughout the hospital stay among twins, although this difference was not statistically significant (OR 2.865, 95% CI 0.598, 13.724, *p* = 0.188). Among singletons, early skin-to-skin contact was significantly associated with an increased likelihood of breastfeeding throughout the hospital stay (OR 3.959, 95% CI 1.153, 13.592, *p* = 0.029), although this association was not significant for twins. Among singletons, no feeding before the first breastfeeding was also associated with an increased likelihood of breastfeeding throughout the hospital stay (OR 11.714, 95% CI 5.038, 27.24, *p* < 0.001), and this relationship was also observed for twins (OR 7.684, 95% CI 2.587, 22.817, *p* < 0.001) (Table 5).

**Table 5:** Factors associated with breastfeeding throughout the hospital stay according to the type of newborn

Type of newborn	Variables	Unadjusted OR (95% CI)	*p*-value	Adjusted OR* (95% CI)	Adjusted *p*-value*
Twins	Feeding before the first breastfeed (no vs. yes)	9.297 (3.325, 25.999)	<0.001	7.684 (2.587, 22.817)	<0.001
	Time to first BMF	0.915 (0.857, 0.977)	0.008	0.916 (0.856, 0.98)	0.011
Singletons	Early skin-to skin contact (yes vs. no)	15.948 (5.52, 46.074)	<0.001	3.959 (1.153, 13.592)	0.029
	Feeding before the first breastfeed (no vs. yes)	11.574 (5.785, 23.156)	<0.001	11.714 (5.038, 27.24)	<0.001
	Time to first BMF	0.891 (0.846, 0.939)	<0.001	0.886 (0.833, 0.942)	<0.001

*BMF* Breastmilk feeding

*Adjusted for mother’s age, residential area, occupation, education, health status, newborn’s birth order, sex, weight, gestational period, Apgar score (after 5 min), and age at discharge

## Discussion

The initiation of early breastfeeding has a significant effect on breastfeeding continuation [17, 31]. Therefore, the present study evaluated factors that influenced breastfeeding throughout the hospital stay among mothers of singleton and twin babies. No feeding before the first breastfeeding was associated with an increased likelihood of breastfeeding throughout the hospital stay, and a delay by each hour in the first breastfeeding session was associated with a lower likelihood of breastfeeding.

The rate of breastfeeding throughout the hospital stay was lower among twins (38.4%), compared to among singletons (46.5%), although the difference was not statistically significant. This result is similar to the findings of a previous study that breastfeeding initiation rates are lower among multiples, compared to among singletons [32]. The present study also found that twins weighed approximately 20% less, compared to singletons, and that twins were more likely to be delivered using a Cesarean section. Twins were much more likely to have a gestational period of ≤ 37 weeks, compared to singletons (71.2% vs. 18.7%). After adjusting for variables that might influence breastfeeding, twins who received early skin-to-skin contact had an increased likelihood of breastfeeding throughout the hospital stay, compared to twins who did not. However, the influence of early skin-to-skin contact was larger among singletons, compared to that among twins. This difference is likely related to the anesthesia’s effects (used during Cesarean section), lower birth weights, and shorter gestational periods among twins.

As the mother undergoes close observation in the delivery room, her newborn immediately receives eye prophylaxis, vitamin K injections, and a physical examination. However, this can create long periods of mother-child separation that preclude breastfeeding, and infants often receive artificial feeding. In addition, mothers of twins may often allow their babies to be artificially fed, based on concerns regarding an insufficient milk supply. This artificial feeding can lead to nipple confusion, and it is important to educate mothers regarding the importance of breastfeeding soon after their delivery.

In the present study, rooming-in was associated with a higher likelihood (albeit not statistically significant) of breastfeeding throughout the hospital stay among twins. However, mothers can often experience embarrassment regarding the care and breastfeeding of twins, and these mothers need a supportive environment to answer their questions regarding latching and other breastfeeding questions. It would also be useful to establish systems to allow these mothers to relax and recover while supporting their ability to breastfeed their infants. For example, a previous study revealed that most mothers of multiples were unable to cope with prolonged periods of rooming-in during the early postpartum period, despite receiving support from family and friends [33]. Thus, a personalized and flexible rooming-in arrangement (in terms of timing and duration) may be useful for promoting breastfeeding throughout the hospital stay among mothers of multiples.

Mothers of twins also experience significant breastfeeding difficulties, and their partner and peers are not effective sources of support [34]. However, the present study did not reveal strong associations between breastfeeding throughout the hospital stay and satisfaction with support from the husband or other family members. This finding may relate to the implementation of a comprehensive breastfeeding support program at the study hospital, which includes personnel education, group education, and a program for breastfeeding supporters.

Interestingly, no feeding before the first breastfeeding exhibited a stronger association with breastfeeding throughout the hospital stay among singletons, compared to among twins. In addition, each hour delay in the first breastfeeding session was more strongly associated with a reduced likelihood of breastfeeding throughout the hospital stay among singletons, compared to among twins. Furthermore, early skin-to-skin contact exhibited a stronger association with breastfeeding throughout the hospital stay among singletons, compared to among twins. The findings of this study suggest that singletons exhibited a higher tendency towards achieving the outcome, and that mothers of twins may experience additional and/or different factors that influence their breastfeeding practice. Therefore, twins should receive their mother’s milk without supplementation (except for medical reason) after early skin-to-skin contact, and start breastfeeding as early as possible to achieve the desired imprint effects. Moreover, mothers of twins typically experience a lack of time (because of the increased number of breastfeeding sessions) as their primary challenge, rather than an inadequate milk supply. The time-consuming nature of breastfeeding multiples may lead to early breastfeeding cessation [10], and it may be preferable to feed the infants individually until they have established breastfeeding, although simultaneous breastfeeding is recommended as a time saving approach after the establishment of breastfeeding [35]. Therefore, obtaining advice from healthcare personnel and from experienced mothers of multiples can help improve the breastfeeding experience [36].

It is also necessary to educate healthcare personnel regarding the importance of breastfeeding and obstacles to breastfeeding [23, 37]. Thus, collaboration among the medical team is necessary to ensure that breastfeeding women receive consistent care and assistance with breastfeeding [20]. Efforts should encourage early breastfeeding, without artificial limits, throughout the hospital stay to improve the breastfeeding rate among mothers of twins.

This study is significant because it investigated factors that influence breastfeeding among mothers of singleton and twin babies throughout their hospital stay. Therefore, this study provides a theoretical basis for interventions to promote effective breastfeeding, and may be useful for designing and implementing breastfeeding education initiatives. Furthermore, this study evaluated the factors that affected breastfeeding among mothers of singletons and twins who received the same care, and the results may be useful for improving nursing practice and education regarding the care for mothers of these types (singleton vs twin) of newborns. The results of the present study provide basic information regarding each type of newborn, suggest direction for policies that aim to improve the breastfeeding rate among the growing number of mothers with multiples, and may ultimately help promote maternal and neonatal health.

However, this study also has several limitations. First, some data were collected retrospectively from hospital records, and the related findings cannot support conclusions regarding the causality of the relationships that were observed. Second, other data were collected with the mothers’ recall, and this technique is associated with important sources of error (e.g., recall bias). Third, the analyses did not consider various emotional and psychological factors that may influence breastfeeding rates. Fourth, this study only evaluated women who delivered their babies at a single institution, and it may be difficult to generalize the results to other populations. Finally, given the low response rate of 20%, we cannot rule out serious selection bias and thus cannot generalize these findings beyond the sample studied.

## Conclusion

The present study evaluated factors that were expected to influence breastfeeding throughout the hospital stay, and aimed to evaluate whether these factors remained significant after adjusting for the mothers’ characteristics, the newborns’ characteristics, and the delivery characteristics. No feeding before the first breastfeeding was more strongly associated with breastfeeding throughout the hospital stay among singletons, compared to among twins. In addition, each hour delay in the first breastfeeding session was more strongly associated with a reduced likelihood of breastfeeding throughout the hospital stay among singletons than among twins. Furthermore, early skin-to-skin contact exhibited a stronger association with breastfeeding throughout the hospital stay among singletons than among twins.

The findings of the present study suggest that singletons exhibited a higher tendency towards achieving the desired outcome. Thus, mothers of twins may experience additional and/or different factors that influence their breastfeeding practice. Therefore, efforts should encourage early breastfeeding, without artificial limits, throughout the hospital stay to improve the breastfeeding rate among mothers of twins. Moreover, an individualized approach that addresses the factors that influence breastfeeding for each newborn may help improve the corresponding rates of breastfeeding throughout the hospital stay.

## Abbreviations

OR: Odds ratio
CI: Confidence interval

## References

[1] Lee GR, Park KH, Park JS, Lee WM, Cha JY, Kim HH (2003). Statistical analysis of twin pregnancy for 10 years (1993–2002). Korean J Obstet Gynecol.

[2] Salihu HM, Aliyu MH, Rouse DJ, Kirby RS, Alexander GR (2003). Potentially preventable excess mortality among higher order multiples. Obstet Gynecol.

[3] Wright VC, Chang J, Jeng G, Chen M, Macaluso M (2007). Centers for Disease Control and Prevention. Assisted reproductive technology surveillance-United States, 2004. MMWR Surveill Summ.

[4] Park SH, Chun DW (2004). Trends of child birth in older maternal age (1985–2003). J Reprod Med Pop.

[5] Health Insurance Review & Assessment Service. 2013. http://www.hira.or.kr/dummy.do?pgmid=HIRAA020041000000&cmsurl=/cms/inform/02/1322002_27116.html&subject. Accessed 11 Dec 2013.

[6] Park SH, Kim JS, Lim DO (2012). Secular trends of age specific multiple birth rate in Korean women; 1998–2010. J Reprod Med Popul.

[7] World Health Organization, UNICEF (2009). Baby-Friendly Hospital Initiative: Revised, updated and expanded for integrated care: Section 3. Breastfeeding promotion and support in a Baby-Friendly Hospital.

[8] Ooki S (2008). Breast-feeding rates and related maternal and infants’ obstetric factors in Japanese twins. Environ Health Prev Med.

[9] Yokoyama Y, Ooki S (2004). Breast-feeding and bottle-feeding of twins, triplets and higher older multiple births. Nihon Koshu Eisei Zasshi.

[10] Damato EG, Dowling DA, Standing TS, Schuster SD (2005). Explanation for cessation of breastfeeding in mothers of twins. J Hum Lact.

[11] Inoue M, Binns CW, Otsuka K, Jimba M, Matsubara M (2012). Infant feeding practices and breastfeeding duration in Japan: A review. Int Breastfeed J.

[12] 12. Minister of Health, Labour and Welfare. In The 2010 National Survey of Child Growth. Edited by Equal Employment Children and Families Bureau. Tokyo: Minister of Health, Labour and Welfare. 2011. [In Japanese]

[13] Kim HR. Trend of breastfeeding in the world and policy direction for promoting breastfeeding. Seoul, Korea Institute for Health and Social Affaires. 2011. http://www.kihasa.re.kr/html/jsp/public/public_01_01.jsp. [In Korean].

[14] Haku M (2007). Breastfeeding: Factors associated with the continuation of breastfeeding, the current situation in Japan, and recommendations for further research. J Med Invest.

[15] Nakao Y, Moji K, Honda S, Oishi K (2008). Initiation of breastfeeding within 120 minutes after birth is associated with breastfeeding at 4 months among Japanese women: A self-administered questionnaire survey. Int Breastfeed J.

[16] Kearney MH, Cronenwett LR, Barrett JA (1990). Breastfeeding problems in the first week postpartum. J Nurs Res.

[17] King RS. Helping Mothers to Breastfeed. Nairobi, AMREF; 1992.

[18] Dabrowski GA (2007). Skin to skin contact: giving birth back to mothers and babies. Nurs Womens Health.

[19] Shaker I, Scott JA, Reid M (2004). Infant feeding attitudes of expectant parents: breastfeeding and formula feeding. J Adv Nurs.

[20] The Joint Commission. Specification manual for Joint Commission national quality Core measures. 2011. http://manual.jointcommission.org/releases/archive/TJC2011A. Accessed 24 May 2011.

[21] Mikiel-Kostyla K (2000). Breastfeeding or twins with regard to pre-term infants. Ginekol Pol.

[22] Gromada KK (1992). Breastfeeding more than one: Multiples and tandem breastfeeding. NAACOGS Clin Issu Perinat Womens Health Nurs.

[23] Hattori R, Hattori H (1999). Breastfeeding twins: Guideline for success. Birth.

[24] Neifert M, Lawrence R, Seacat J (1995). Nipple confusion: Toward a formal definition. J Pediatr.

[25] Bennington LK (2011). Breastfeeding multiples: It can be done. Newborn Infant Nurs Rev.

[26] Neville MC, Morton J, Umemura S (2001). Lactogenesis. The transition from pregnancy to lactation. Pediatr Clin North Am.

[27] Helsing E (1985). Infant feeding and infectious illness. Report of the World Health Organization.

[28] Gray-Donald K, Kramer MS, Munday S, Leduc DG (1985). Effect of formula supplementation in the hospital on the duration of breast-feeding: A controlled clinical trial. Pediatrics.

[29] Sakong P, Kim EK, Kim Y, Kim YI, Lee JS (2009). Factors affecting breastfeeding initiation; Analysis by time sequences after delivery. J Korean Soc Matern Child Health.

[30] Bendel RB, Afifi AA (1977). Comparison of stopping rules in forward regression. J Am Stat Assoc.

[31] Kim BY, Kim JH (2013). Influence of an early latching-on program on the breastfeeding rate. Perspectives in Nursing Science.

[32] Geraghty SR, Khoury JC, Kalkwarf HJ (2004). Comparison of feeding among multiple birth infants. Twin Res.

[33] Auer C, Gromada KK (1998). A case report of breastfeeding quadruplets: Factors perceived as affecting breastfeeding. J Hum Lact.

[34] Guyer J, Millward LJ, Berger I (2012). Mothers’ breastfeeding experiences and implications for professionals. Br J Midwifery.

[35] Flidel-Rimon O, Shinwell ES (2006). Breast feeding twins and high multiple. Arch Dis Child Fetal Neonatal Ed.

[36] Cinar ND, Alvur TM, Kose D, Nemut T (2013). Breastfeeding twins: A qualitative study. J Health Popul Nutr.

[37] LaFleur EA, Niesen KM (1996). Breastfeeding conjoined twins. J Obstet Gynecol Neonatal Nurs.

